# An intelligent decision support system for acute postoperative endophthalmitis: design, development and evaluation of a smartphone application

**DOI:** 10.1186/s12911-023-02214-3

**Published:** 2023-07-21

**Authors:** Mahdi Shaeri, Nasser Shoeibi, Seyedeh Maryam Hosseini, Fatemeh Rangraze Jeddi, Razieh Farrahi, Ehsan Nabovati, Azam Salehzadeh

**Affiliations:** 1 Department of Ophthalmology, Kashan University of Medical Sciences, Kashan, Iran; 2 Eye Research Center, Mashhad University of Medical Sciences, Mashhad, Iran; 3 Health Information Management Research Center, School of Allied Health Professions, Kashan University of Medical Sciences, Pezeshk Blvd, 5Th of Qotbe Ravandi Blvd - Pardis Daneshgah, Kashan, 8715973449 Iran; 4 Department of Health Information Technology, Ferdows Faculty of Medical Sciences, Birjand University of Medical Sciences, Birjand, Iran

**Keywords:** Eye disease, Endophthalmitis, Clinical decision support, Mobile application, Artificial intelligence, User-centered design

## Abstract

**Background:**

Today, clinical decision support systems based on artificial intelligence can significantly help physicians in the correct diagnosis and quick rapid treatment of endophthalmitis as the most important cause of blindness in emergency diseases. This study aimed to design, develop, and evaluate an intelligent decision support system for acute postoperative endophthalmitis.

**Methods:**

This study was conducted in 2020–2021 in three phases: analysis, design and development, and evaluation. The user needs and the features of the system were identified through interviews with end users. Data were analyzed using thematic analysis. The list of clinical signs of acute postoperative endophthalmitis was provided to ophthalmologists for prioritization. 4 algorithms support vector machine, decision tree classifier, k-nearest neighbors, and random forest were used in the design of the computing core of the system for disease diagnosis. The acute postoperative endophthalmitis diagnosis application was developed for using by physicians and patients. Based on the data of 60 acute postoperative endophthalmitis patients, 143 acute postoperative endophthalmitis records and 12 non-acute postoperative endophthalmitis records were identified. The learning process of the algorithm was performed on 70% of the data and 30% of the data was used for evaluation.

**Results:**

The most important features of the application for physicians were selecting clinical signs and symptoms, predicting diagnosis based on artificial intelligence, physician–patient communication, selecting the appropriate treatment, and easy access to scientific resources. The results of the usability evaluation showed that the application was good with a mean (± SD) score of 7.73 ± 0.53 out of 10.

**Conclusion:**

A decision support system with accuracy, precision, sensitivity and specificity, negative predictive values, F-measure and area under precision-recall curve 100% was created thanks to widespread participation, the use of clinical specialists' experiences and their awareness of patients' needs, as well as the availability of a comprehensive acute postoperative endophthalmitis clinical dataset.

**Supplementary Information:**

The online version contains supplementary material available at 10.1186/s12911-023-02214-3.

## Background

Endophthalmitis is one of the most important causes of blindness in emergency eye diseases [1, 2]. Endophthalmitis affects the inner layers of the eye and is associated with substantial and progressive inflammation of the vitreous [3]. Acute postoperative endophthalmitis (APE) is a type of exogenous endophthalmitis that usually occurs after any invasive eye surgery, and is the most devastating complication after intraocular procedures [4]. With an incidence rate of 0.04–4% worldwide, this type of endophthalmitis is the most common type of the disease, and in 90% of cases, the disease occurs after cataract surgery [4]. A study conducted in an Eye Institute showed that the incidence of endophthalmitis after cataract surgery was 0.09% in a 10-year period [5]. The results of a review article conducted in Iran from January 2015 to February 2016 show that the incidence of endophthalmitis after cataract surgery is 0.02–0.1% [6]. In case of early diagnosis of endophthalmitis, the destruction of the eye structure can be somewhat prevented to preserve vision. Therefore, early diagnosis and proper treatment are critically important [7, 8].

Mobile health has created new opportunities to provide healthcare services. The need for quick access to information, physicians’ correct diagnosis and early action based on clinical guidelines, and patients’ early presentation has led to the use of this technology by physicians and other healthcare staff. This technology has been increasingly used by medical specialists, including ophthalmologists, in clinical and educational settings [9, 10].

In the last decade, ophthalmologists have used electronic health record systems, clinical decision support systems (CDSSs), office management, and video consultations through smartphones and tablets to diagnose and treat eye diseases [11, 12]. The smartphone-based intelligent decision support system is a type of mobile health platform that assists ophthalmologists in diagnosis and treatment, and as a result, increases the accuracy of diagnosis and reduces treatment costs. Romero et al., (2019) designed a decision support system based on fuzzy rules to help diabetic retinopathy (DR) screening programs [13]. The OphthalDSS decision support application was designed for the diagnosis of the red eye at the University of Valladolid, Spain. This application is capable of diagnosing over 30 eye diseases and can be used as an educational tool [14].

Few clinical computer systems are currently used in ophthalmology, mostly as a guideline for eye diseases or for teaching ophthalmic surgical skills [15]. The biggest challenge and error in the ophthalmology emergency department, from an ophthalmologist’s perspective, is related to the ability to distinguish endophthalmitis from common postoperative inflammations in a timely manner [16, 17]. To the best of our knowledge, this study is the first of its kind to design a smartphone-based CDSS to assist physicians with early diagnosis of endophthalmitis and also help patients present to medical centers early so as to improve the quality of care and prevent complications of the disease.

## Methods

### Setting

The study was conducted with the participation of a multi-specialty team from Khatam-Al-Anbia Eye Hospital, Mashhad and Kashan University of Medical Sciences in 2020–2021. Khatam-Al-Anbia Eye Hospital is the only educational, research, and healthcare center affiliated with Mashhad University of Medical Sciences and the ophthalmology hub of Northeast Iran. This hospital has 64 licensed beds, 11 specialized clinics, para clinical units (angiography, optometry, imaging and laboratory), 19 operating rooms, 32 recovery beds and a pediatric anesthesia room. The hospital provides ophthalmology specialty and subspecialty services to 250,000 patients annually.

### Study design

The study was conducted in three phases of analysis, design and development, and evaluation.

### Research and development team

The research team included ophthalmologists (retina fellows), health information technology and medical informatics specialists, fellow assistants, and second and third year ophthalmology residents. In the first phase, six ophthalmologists (retina fellows), five health information technology and medical informatics specialists, and five fellow assistants and ophthalmology residents participated. In the second phase, two application designers were added to the team. In the third phase, all the second and third year ophthalmology residents (*n* = 18) and an ophthalmologist working in the emergency room were enrolled.

### Study phases

#### Needs assessment and analysis

##### Literature review

In order to identify the clinical signs of APE and also valid clinical guidelines for the disease, all related sources including books (the 9th and 12th Editions of the American Academy of Ophthalmology [18, 19]), articles, reputable websites such as the website of the Ministry of Health of Iran [20], and the website of the American Academy of Ophthalmology [21], Endophthalmitis Vitrectomy Study [22], and the clinical guidelines of the European Society of Cataract and Refractive Surgeons [23] were reviewed for the prevention and treatment of endophthalmitis after cataract surgery. To determine the features of the decision support application, articles published between 2010 and 2020 and indexed in PubMed, Scopus and Web of Science as well as websites and similar CDSSs were examined to prepare an initial list of their functional features.

##### Interviews

To determine the needs and expectations of users and the content of the application, several semi-structured interviews based on the interview guide were conducted with ophthalmologists from April, 2021 to June, 2021(Supplementary file 1: Table S1). Arrangements were made to invite ophthalmologists (retina fellows) and residents for an interview in person. The interviews were conducted in a comfortable place, and each interview lasted for an average of 40–120 min. Interviews were audio-recorded and key notes were taken by the interviewer. The participants gave their informed consent to participate and the interviews were audio-recorded if they consented to. Also, the participants were assured about the confidentiality of the information and the anonymous publication of the study results. After the completion of each interview, the recorded interview was transcribed and the concepts were coded. Due to the small number of participants, the analysis of conceptual codes and themes was done manually. Data saturation was achieved after completion of 11 interviews.

##### Analysis

**Fig. 1:**
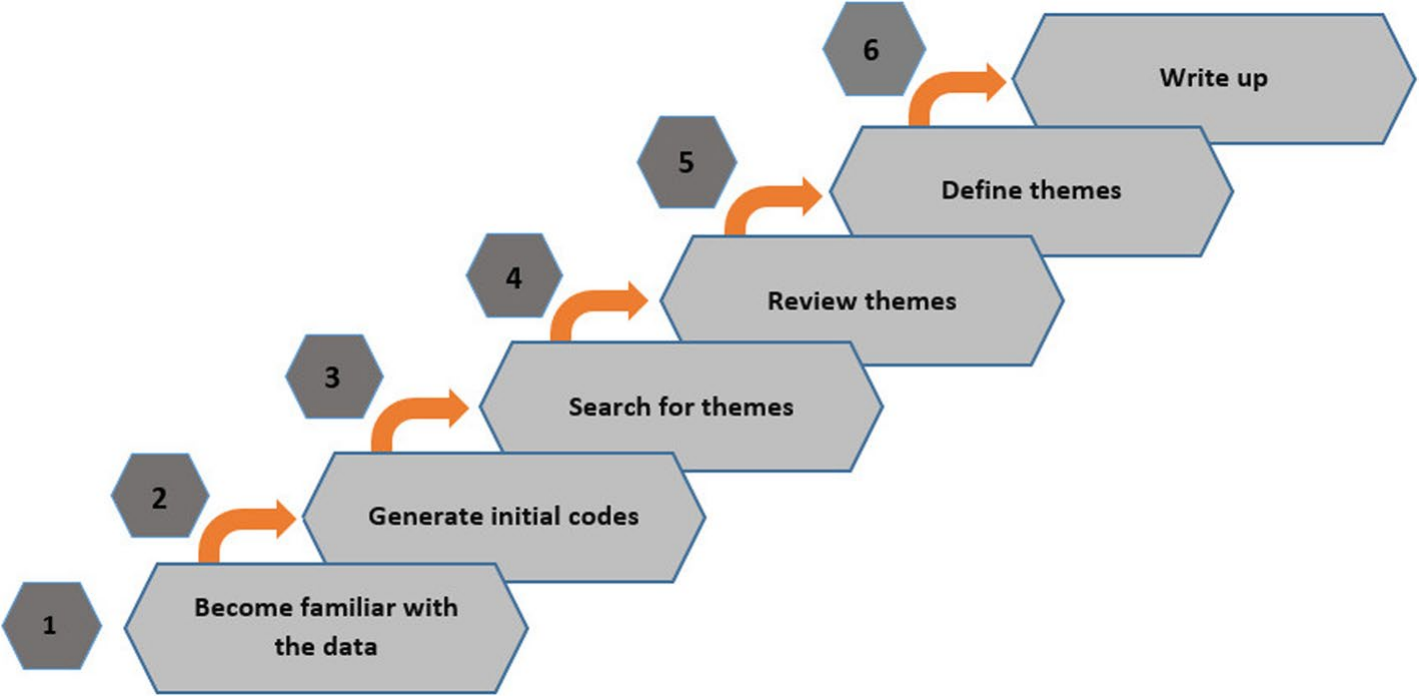
Braun and Clarke’s six-step thematic analysis [24]

Braun and Clarke's thematic analysis was used to investigate the content of the interviews. This analysis allows continuous back-and-forth among datasets and code sets, and analysis of the generated data (Fig. 1) [24].

Based on this method, the interviews audio-recorded by the researcher were first transcribed. The interviews were reread and rewritten to become more familiar with the data. After extracting the basic concepts from the transcripts, two evaluators performed open coding independently (coded by evaluator two and reviewed by evaluator one) and subthemes were drawn. For example, several concepts (codes) were related to drug dosage and providing therapeutic procedures for endophthalmitis, which were merged into a subtheme called selecting the appropriate treatment. Two or more subthemes were merged to form the main theme, and the themes were reviewed again to present the features that ophthalmologists needed. The transcripts of all interviews were examined and coded using Microsoft Word 2016. The desired technical features of the application were also identified in the focus group meeting attended by health information technology and medical informatics specialists. Then, the final list of features was prepared by merging of the features drawn from the interview and throughout the focus group meeting.

#### Design and development

##### Constructing model

**Data collection**

The list of clinical signs of APE was prepared based on scientific sources and clinical guidelines and provided to retina fellows for prioritization and weighting. The list of clinical signs was completed based on the clinical data of 60 patients with endophthalmitis admitted to the study hospital from December 2018 to June 2020.

**Data preparation**

Through the process of data preparation and cleaning processes, Missing data were replaced by multiplying the median of each feature by the weight obtained for each feature which was determined by each specialist. Because clinical signs were multivariate, after pre-processing and converting them to univariate, a number of 155 records were obtained. Out of 155 records,143 APE records and 12 non-APE records remained for further analysis.

**Data partition**

Since the number of data was too small to detect the presence or absence of the disease with the application, machine learning was needed. Therefore, 4 algorithms was used to diagnose the disease to determine the most appropriate algorithm with the best performance.

We used Support Vector Machine (SVM), Decision Tree (DT), K-Nearest Neighbors (KNN) and Random Forest (RF) algorithms to build a classifier using training data.

The following machine learning algorithms are used for classification and regression problems [25]. The codes of each algorithm were used from the corresponding class of the scikit-learn library available in the Python programming language.

*SVM*: SVM is an extension of the support vector classifier and is obtained by extending the feature space in a specific way, using the kernel [26]. Here, the radial kernel with default parameter is used to classify the patients into two categories, "disease-affected" and "disease-free".

*DT*: A decision tree is a set of conditions organized in a hierarchical structure. It is a predictive model in which an instance is classified by following the path satisfying the conditions from the root of the tree until it reaches a leaf, which will correspond to a class label. This algorithm is formed in the form of a decision tree by examining the characteristics of the data and making decisions based on them, and is applied to each conditional branch that is placed on the characteristics of the data to predict the output [25]. decision_tree (X_Train, Y_Train): This function builds a decision tree model using the CART method on the training data and draws its graph. It then returns the model as output.

*KNN*: In pattern recognition, the KNN algorithm is an instance-based learning method used to classify objects based on their closest training examples in the feature space [25]. In this method, three of the nearest neighbors (K = 3) for each subject were discovered in the entire training dataset (APE and non-APE). According to the label of these neighbors, which indicates how many of these three people are not healthy and how many are healthy, the most frequent label can be considered for the person under examination.

*RF*: An RF classifier consists of a number of trees, each of which is grown using some form of randomness. In the standard tree, each node is split using the best distribution among all the variables while in RF each node is split using the best distribution among the predictors in a way random [25].

RF is essentially a set of associative DTs where each voting tree for the class is assigned to a given sample, with the most frequent answer winning the vote [27].

To this end, the dataset of this study, consisting of 143 APE records and 12 non-APE records, was divided into two groups, the training dataset, and the testing dataset. The learning process of the algorithm was performed on 70% of the data (*n* = 108) in the training dataset, and performance evaluation of the algorithm was performed using 30% of the data (*n* = 47) in the testing dataset.

**Evaluation of the model performance**

The effectiveness of the application was investigated using the confusion matrix. The classification and diagnosis of individuals with APE using confusion matrix analyses produced four cases: true positives (TP), true negatives (TN), false positives (FP), and false negatives (FN). By using a confusion matrix, the four classification indices accuracy, precision, sensitivity, and specificity were calculated (Table 1).

**Table 1:** Common metrics in model evaluation [28]

Evaluation metrics	Definitions	Formula
Accuracy	Percentage of correct identification of both positive and negative results. The higher the accuracy, the better the classifier	$\frac{\mathrm{TP}+\mathrm{TN}}{\mathrm{TP}+\mathrm{TN}+\mathrm{FP}+\mathrm{FN}}$
Precision/Positive Prediction Value (PPV)	Proportion of correctly identified positives out of all samples identified as positive	$\frac{\mathrm{TP}}{\mathrm{TP}+\mathrm{FP}}$
Sensitivity/True Positive Rate (TPR)	Proportion of correctly identified positives	$\frac{\mathrm{TP}}{\mathrm{TP}+\mathrm{FN}}$
Specificity/True Negative Rate (TNR)	Proportion of correctly identified negatives	$\frac{\mathrm{TN}}{\mathrm{TN}+\mathrm{FP}}$
Negative Predictive Values (NPV)	Proportion of correctly identified negatives out of all samples identified as negative	$\frac{\mathrm{TN}}{\mathrm{TN}+\mathrm{FN}}$
F-measure	Harmonic mean of precision and sensitivity. The highest F1 score is 1 and the lowest is 0	$\frac{2 *\mathrm{ TP}}{(2 *\mathrm{ TP }+\mathrm{ FP}+\mathrm{FN})}$

##### CDSS design

The user interface of the decision support application was designed based on the functional features determined by Microsoft Visio Drawing 2016. The user interface was presented to the interviewees through face-to-face meetings for re-examination, and then based on their comments, final modifications were made to it. The user interface designed in Microsoft Visio Drawing was also provided to the programmer for coding.

#### Usability evaluation

After registering as a hypothetical physician and patient, the procedure of working with different parts of the APE Dx were explained to the residents in person. Then the application was reviewed by 19 ophthalmologists for two weeks. After completion of the reviews, the Questionnaire for User Interface Satisfaction (QUIS) [29] whose validity [30, 31] and reliability had previously been confirmed [32] was filled out for usability evaluation. QUIS consists of five main domains with 27 items rated on a 10-point (0–9) Likert scale (Supplementary file 2: Table S2). To analyze the data on usability evaluation, the mean (± standard deviation (SD)) scores on each domain was first calculated, and then the scores were classified into three levels of poor [0–3], average [3-6] and good (6–9].

## Results

### Needs assessment and analysis

In the focus group meeting attended by six retina fellows, consensus was achieved to refer to the 12th Edition of the American Academy of Ophthalmology [19] and the European Society of Cataract and Refractive Surgeons [23] for the clinical guideline of endophthalmitis. The clinical signs of the disease drawn from the literature review were prioritized and weighted (from 1 to 10) by the experts (Table 2).

**Table 2:** Average weight and priority of clinical signs of endophthalmitis

Priority	Signs	Average
1	Eye Pain	7.8
2	Red Eyes	6.5
3	Marcus Gunn Pupil	9.7
4	Hypopyon	9.5
5	Visual Acuity	7
6	B Scan	7.5
7	Lid Swelling	5.8
8	Fibrin in AC	6
9	AC Cell	7.2
10	Red Reflex	8.7
11	Vitreous Cell	8.8
12	Eye Discharge	4.3
13	Chemosis	4.8
14	Corneal Involvement	9.5

The interviewees participated in this phase were five residents and six faculty members (nine men and two women). The thematic analysis of the interview transcripts yielded 18 primary themes and 9 subthemes, and finally two main themes were generated by the evaluators (Supplementary file 3: Table S3).

The results from the literature review and interviews yielded seven functional features for physicians and five functional features for patients in the application (Table 3).

**Table 3:** Application’s functional features identified for physicians and patients

Functional features	
Physician	Patient
Selecting clinical signs and symptoms Predicting diagnosis based on artificial intelligence Physician to patient communication Selecting the appropriate treatment Easy access to scientific resources Summary and reporting Assigning the English language	Playing audio files Uploading images Sharing data with physicians patient to physician communication Education (Access to clinical information) Assigning the Persian language

### The application design and development phase

The APE clinical decision support application (*APE Dx*) was designed based on the functional features determined in the back-end and front-end modules. The artificial intelligence algorithm was coded with Python programming language. The front-end module of the physician–patient user interface was written using *PWA* and *jQuery*, and the back-end module was written using *Django*. The application is web-based and available anywhere any time through all browsers and operating systems. After designing the initial version, the application link was provided to ophthalmologists for review and approval. The application bugs identified by the experts were fixed and the final version was developed.

**Fig. 2:**
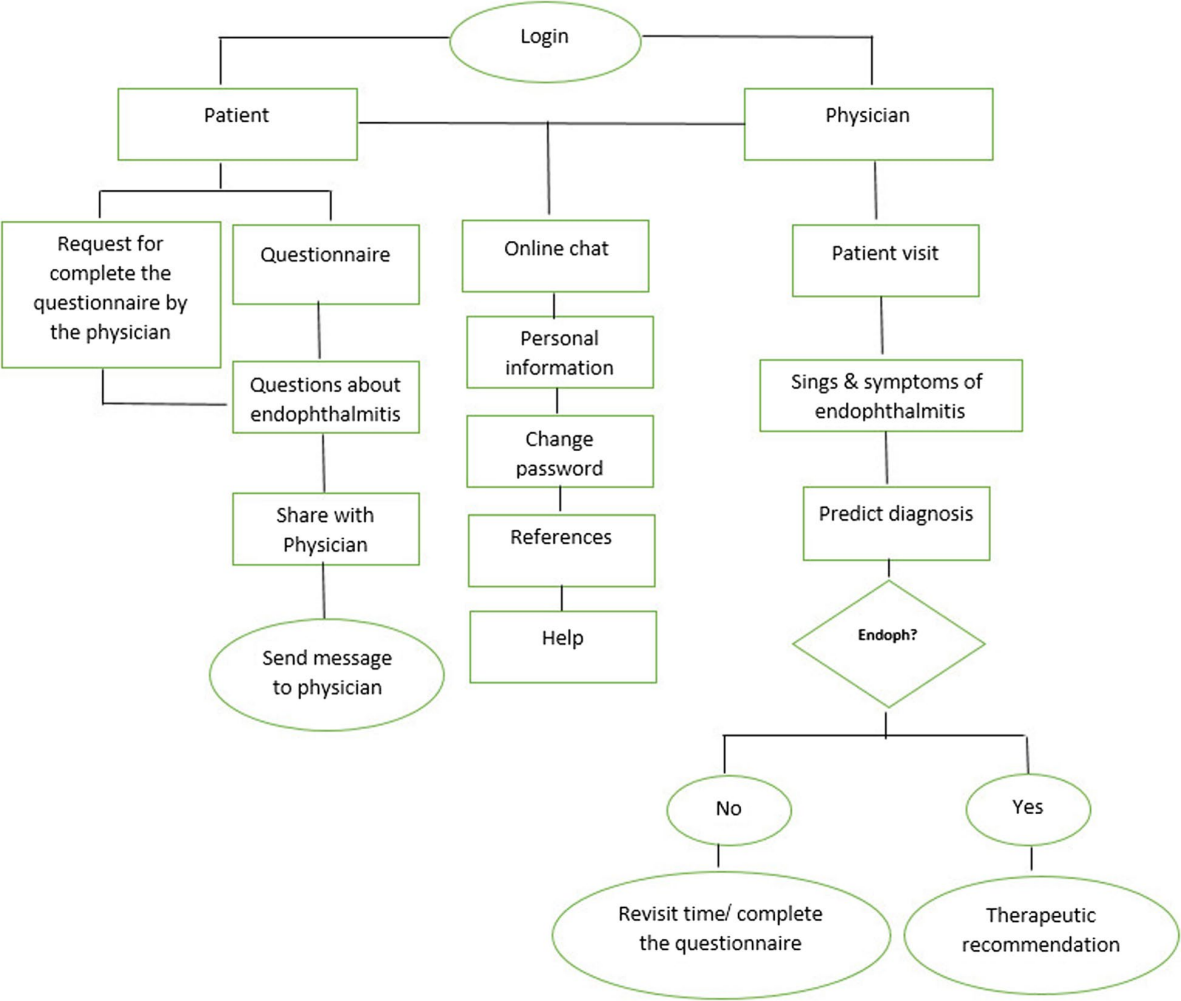
The main components of physician–patient user interface

**Fig. 3:**
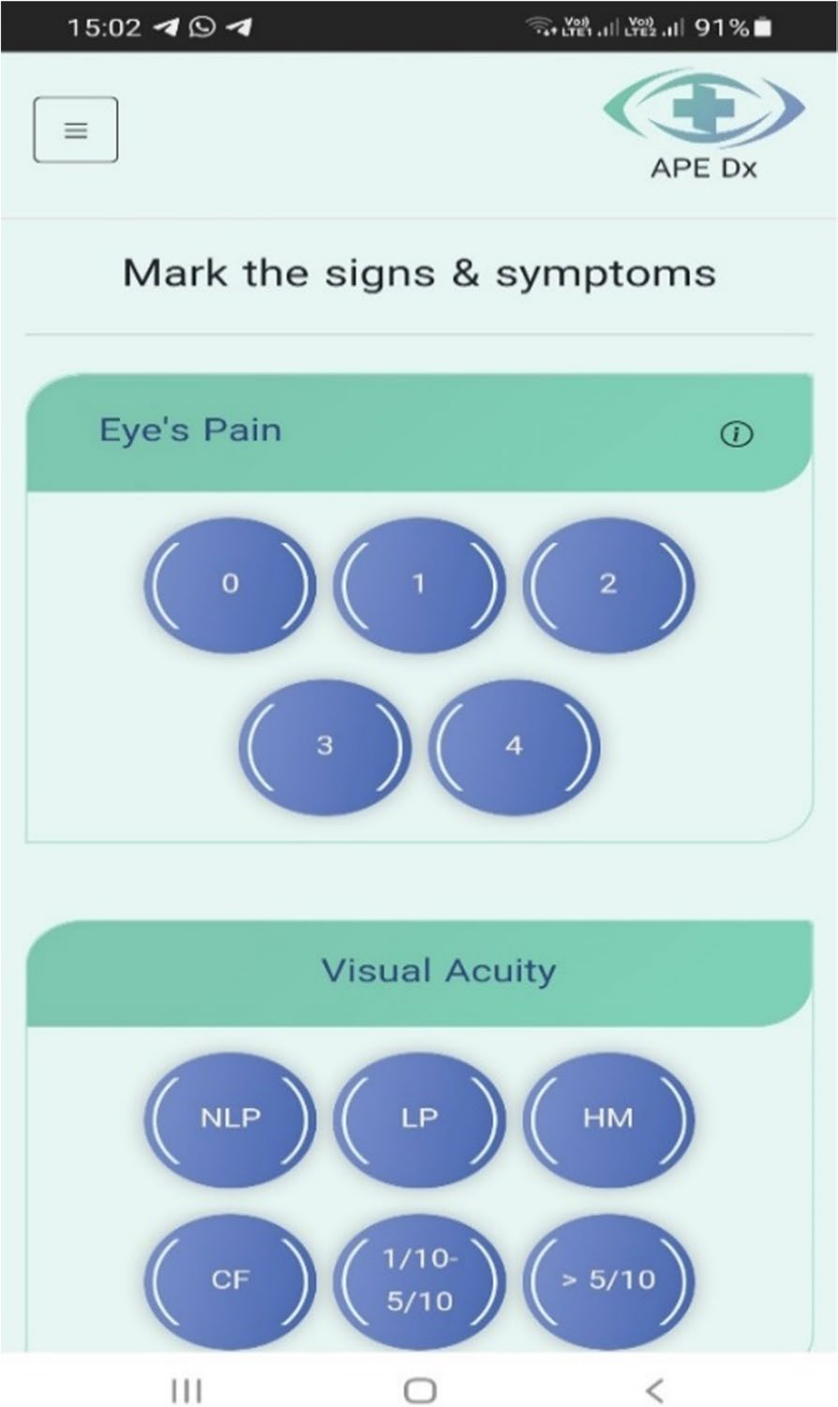
Signs & symptoms from physician interface

**Fig. 4:**
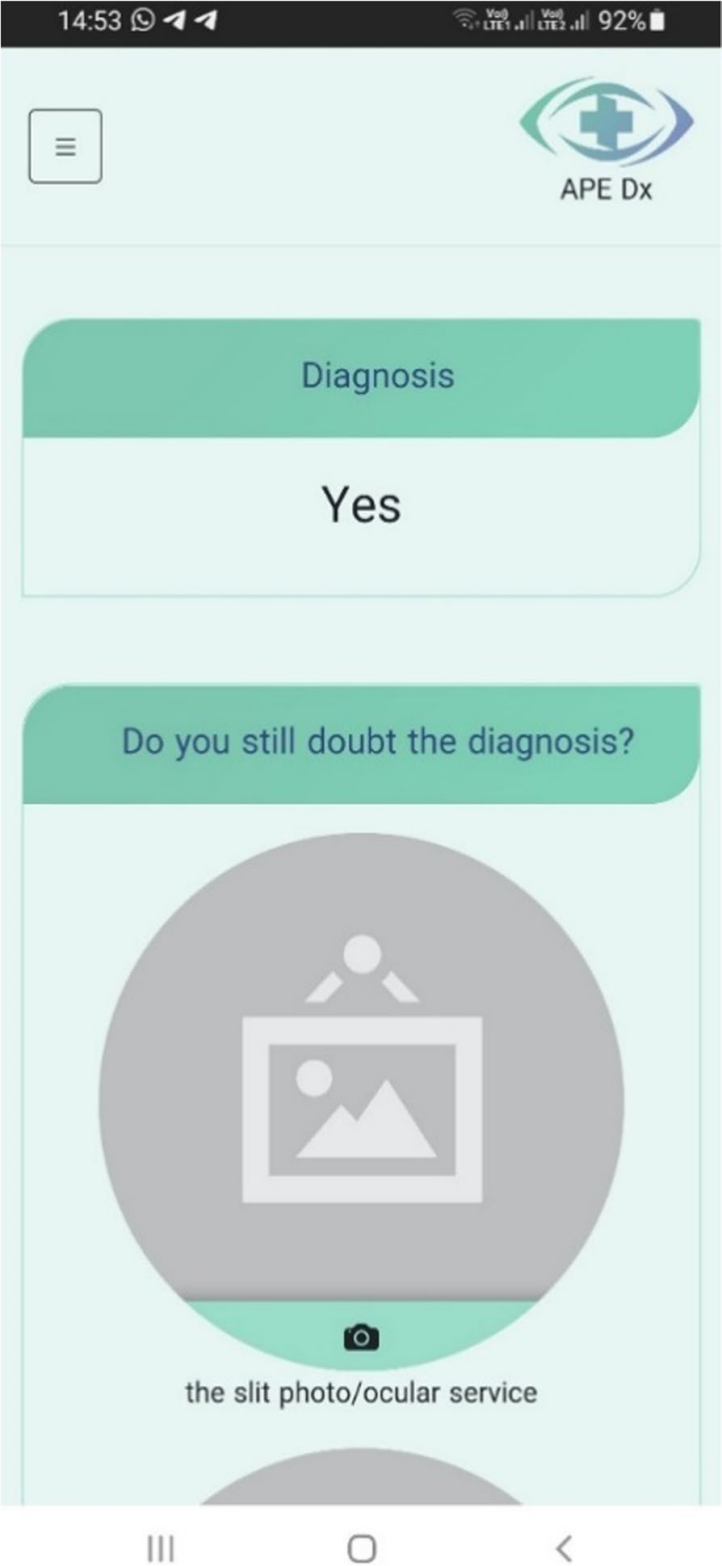
Diagnosis from physician interface

**Fig. 5:**
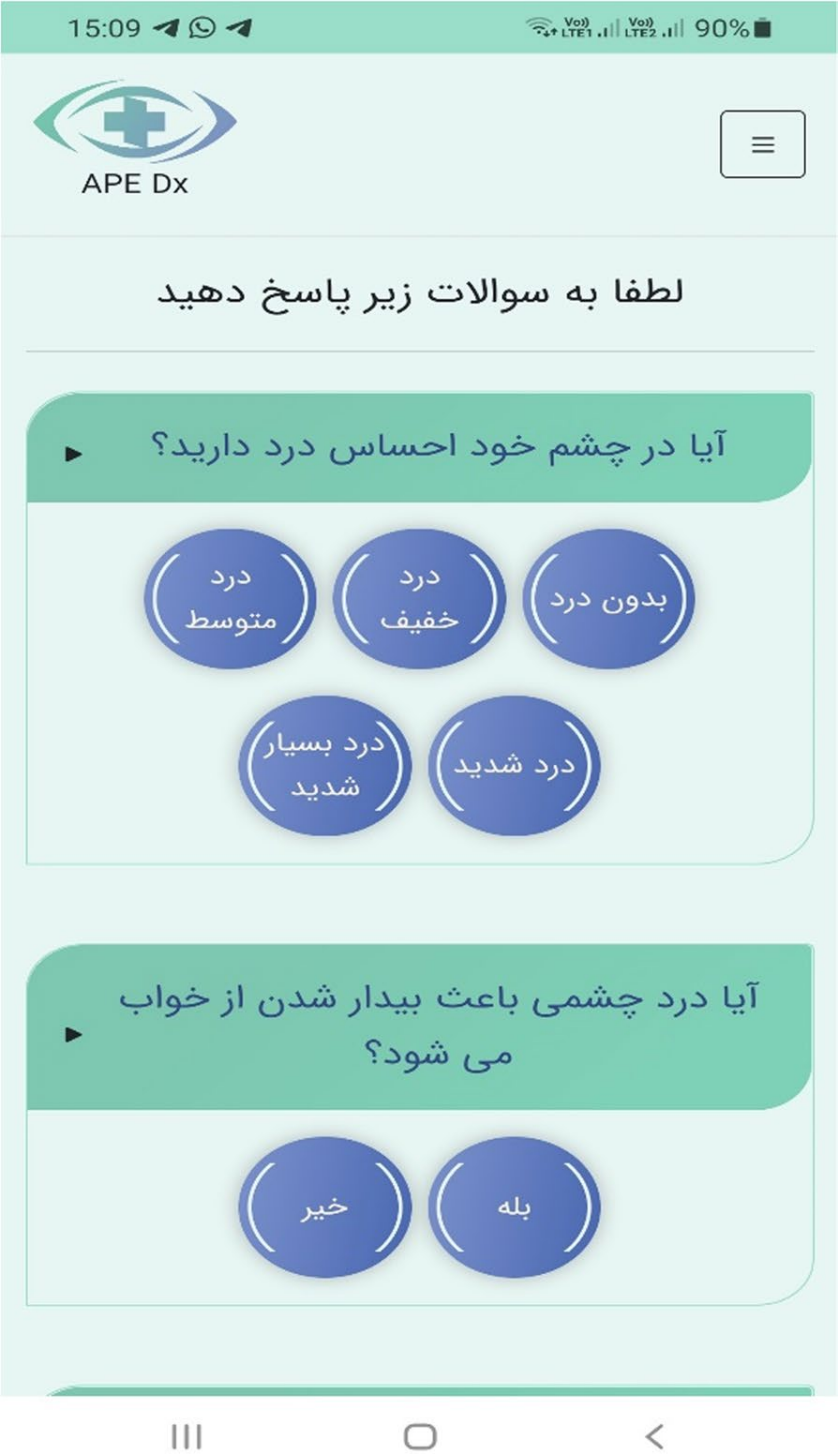
Patient questionnaire from patient interface

**Fig. 6:**
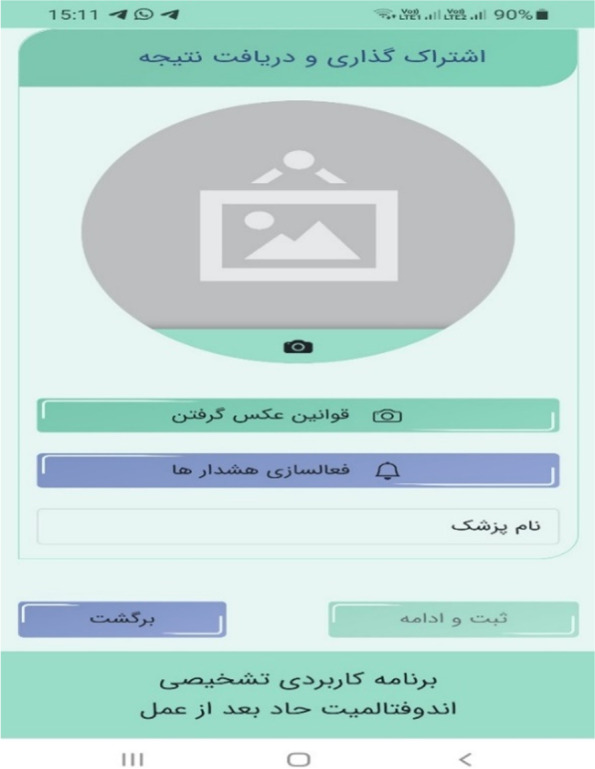
Information sharing from patient interface

The main components of the physician–patient user interface are thematically illustrated in Fig. 2 and screenshots of the decision support application in Figs. 3, 4, 5 and 6. (Supplementary file 4: Fig. S1).

### Evaluation of the application

The criteria calculated in this research include: accuracy, precision, sensitivity, specificity, NPV and F-measure.

This criteria of the application were calculated by confusion matrix (Table 4).

**Table 4:** Confusion matrix

Label	Endophthalmitis	Non-Endophthalmitis	Total
Endophthalmitis	42 (TP)	0 (FP)	42
Non-Endophthalmitis	0 (FN)	5 (TN)	5
Total	42	5	47

$$\mathrm{Accuracy }=\frac{\mathrm{TP}+\mathrm{TN}}{\mathrm{TP}+\mathrm{TN}+\mathrm{FP}+\mathrm{FN}}=\frac{147}{47}= 1$$

$$\mathrm{Precision }=\frac{\mathrm{TP}}{\mathrm{TP}+\mathrm{FP}}= \frac{42}{42+0}= 1$$

$$\mathrm{Sensitivity }=\frac{\mathrm{TP}}{\mathrm{TP}+\mathrm{FN}}= \frac{42}{42+0}= 1$$

$$\mathrm{Specificity }=\frac{\mathrm{TN}}{\mathrm{TN}+\mathrm{FP}}=\frac{5}{5+0}= 1$$

$$\mathrm{NPV}=\frac{\mathrm{TN}}{\mathrm{TN}+\mathrm{FN}}=\frac{5}{5+0}= 1$$

$$\mathrm{F}-\mathrm{measure}=\frac{2 * \mathrm{T}\mathrm{P}}{(2 * \mathrm{T}\mathrm{P} + \mathrm{F}\mathrm{P}+\mathrm{F}\mathrm{N}) }=\frac{2 *42}{(2 * 42 + 0+0) }= 1$$

The findings showed in all 4 reviewed algorithms that the decision support application's sensitivity was calculated at 100%, meaning that it could properly diagnose 100% of participants with APE. The application's specificity was determined to be 100%, meaning that it could properly classify 100% of the investigated individuals as non-APE patients even though they did not have the disease. The application successfully distinguished 100% of disease cases from true and false positive cases when the precision was calculated at 100. Overall, 100% of instances with and without APE could be correctly diagnosed by the application. Considering accuracy and sensitivity, the F-measure criterion was obtained 1, which indicates the high accuracy of this decision support system in diagnosing the disease.

In addition, the Receiver Operator Characteristic (ROC) diagram and the Precision-Recall (sensitivity) diagram are drawn, which show how well the machine learning model performs for the diagnosis of two categories sick and healthy.

**Fig. 7:**
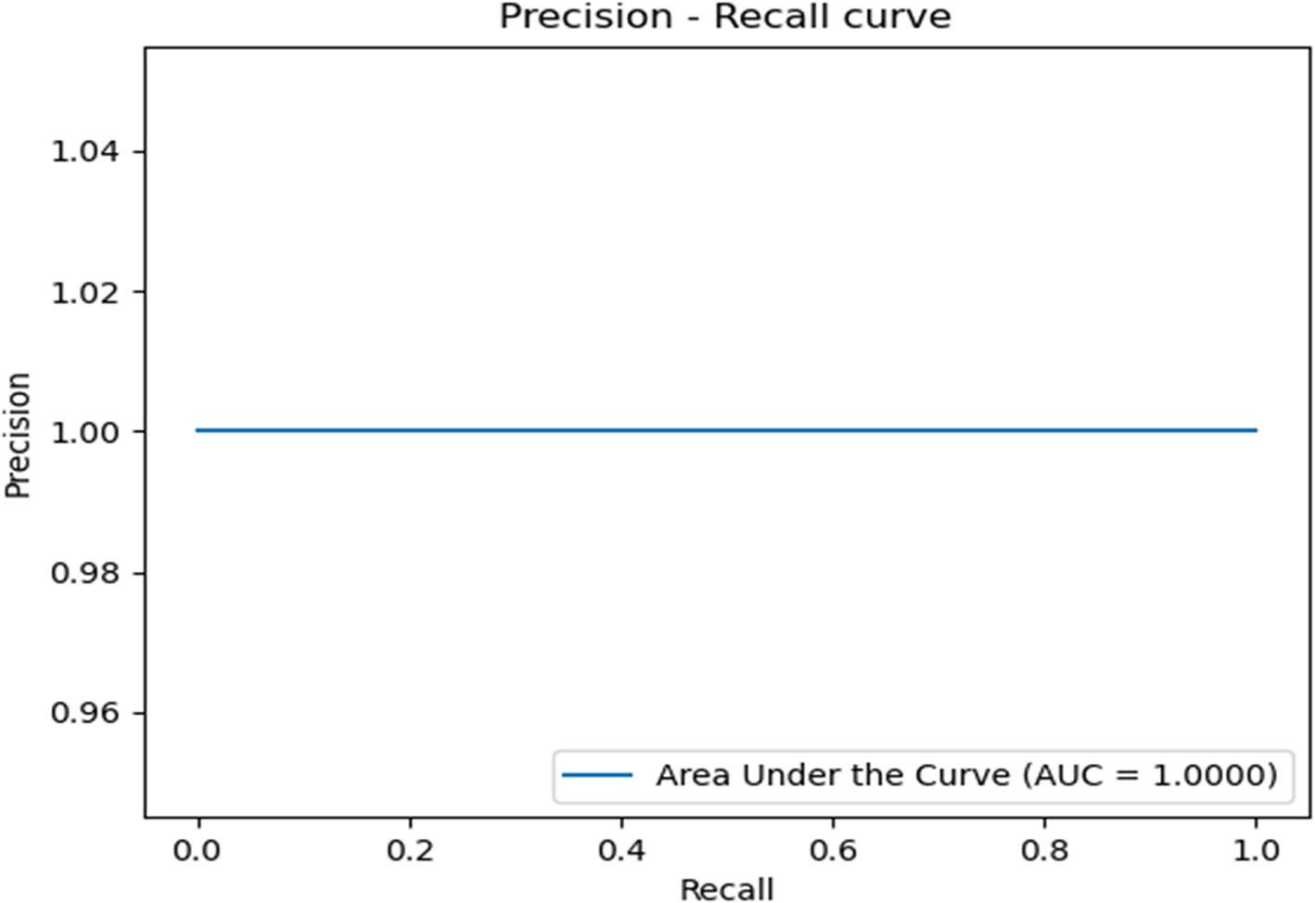
Precision- recall curve

The same accuracy curve was obtained for all 4 algorithms (Fig. 7).

The ROC curve is widely used to show the performance of a model or algorithm. The ROC curve provides performance information across a range of thresholds and can be summarized by the area under the curve (AUC), a single number [33]. Typically, an ROC analysis shows how sensitivity (true positive rate) changes with varying specificity (true negative rate or 1 – false positive rate) for different thresholds.

**Fig. 8:**
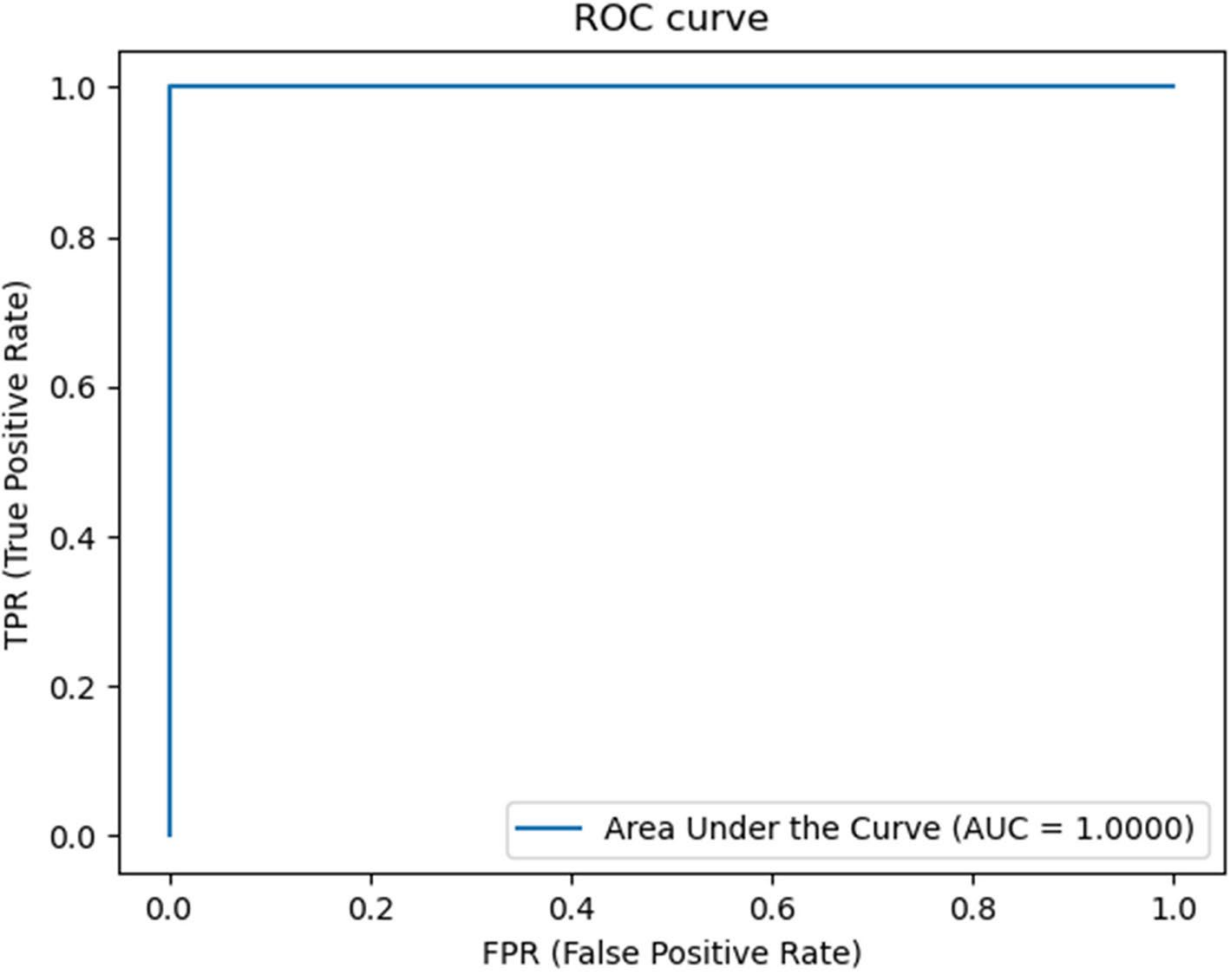
ROC curve

The curve was the same for all 4 algorithms and the value of AUC was obtained 1 (Fig. 8).

The dataset was divided into two categories: healthy with 0 label and patient with 1 label. By evaluating the datasets with four algorithms, the performance evaluation criteria values of each model were similar to each other. The reason that all criteria are 100% is that the dataset of healthy people is zero and the dataset of patients has values greater than zero, so all algorithms can easily distinguish between healthy and sick.

**Fig. 9:**
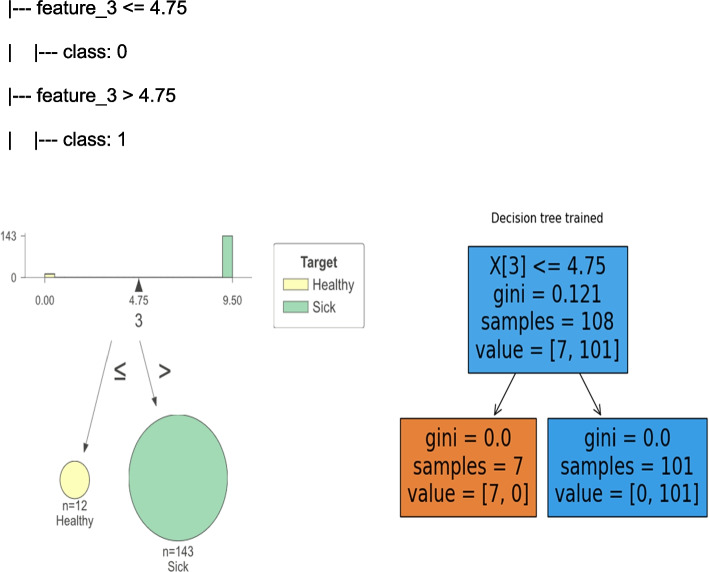
Decision tree trained

According to the obtained results, all the algorithms of this study have worked successfully and all the evaluation criteria show very high precision. Therefore, to choose the best algorithm for this decision support system, the range and type of data, data volume, speed and efficiency, and specific needs should be considered. According to the opinion of experts, a decision tree is one of the best classifiers due to its simple interpretation and expressive quality [34]. Therefore, the decision tree algorithm was used in the decision support system (Fig. 9).

The demographic characteristics of the residents participating in usability evaluation are shown in Table 5.

**Table 5:** Demographic characteristics of users participating in usability evaluation

Demographic characteristics	Frequency	Percentage
Age (year)		
≤ 29	9	47.4
≥ 30	10	52.6
Gender		
Male	10	52.6
Female	9	47.4
Education level		
Second year resident	7	36.8
Third year resident	11	57.9
Emergency ophthalmologist	1	5.3
Work experience as an ophthalmologist (year)		
2	7	36.8
3	11	57.9
3 <	1	5.3

The results of the usability evaluation showed that the application was *good* with a mean (± SD) score of 7.73 ± 0.53 and obtained the highest score on the learning and screen domain (Table 6).

**Table 6:** The results of usability evaluation of the *APE Dx* application

Domains	Mean (± SD)
Overall reaction to the software	7.14 ± 0.55
Screen	8.13 ± 0.66
Terminology and system information	7.66 ± 0.44
Learning	8.26 ± 0.5
System capabilities	7.44 ± 0.54
Total	7.73 ± 0.53

## Discussion

In this study, an intelligent clinical decision support application (APE Dx) was designed and developed for APE diagnosis. The most important features of this application for physicians were, respectively, selecting clinical signs and symptoms, predicting diagnosis based on artificial intelligence, physician–patient communication, selecting the appropriate treatment, easy access to scientific resources, summary and reporting, and assigning the English language. The most important features for patients were, respectively, playing audio files, uploading images, sharing data with physicians, patient-physician communication, education (access to clinical information), and assigning the Persian language. The accuracy, precision, sensitivity, specificity, NVP, F-measure and AUC of the application were obtained 100%. The usability evaluation of the application was obtained as *good* by the residents, as well.

Artificial intelligence algorithm was used in the APE Dx decision support application to more accurately diagnose the disease based on clinical signs. Artificial intelligence algorithm was used in our study for the diagnosis of eye diseases, which is consistent with other studies which designed a CDSS based on fuzzy random forest and a set of fuzzy decision trees for the diagnosis and treatment of diabetic retinopathy [13], Deep Neural Networks (DNNs) and Boltzmann Machines model for the early diagnosis of hypertensive retinopathy [35], artificial neural network algorithm for retina image analysis [36], and complex image analysis and machine learning techniques for the early screening of diabetic retinopathy [37]. Artificial intelligence techniques can support medical diagnosis processes, increase the accuracy of diagnosis, and be used in designing appropriate models to identify affected people and predict diseases [38]. In this study, we used 4 artificial intelligence algorithms including SVM, DT, KNN and RF to predict disease diagnosis. Since, to the best of our knowledge, CDSS based on artificial intelligence algorithms in ophthalmology have so far focused on diabetic retinopathy, it is recommended that artificial intelligence algorithm-based CDSS be used for accurate diagnosis and provision of appropriate treatment for other eye diseases, as with the current study, with regard to setting conditions, expert opinions and available data.

In the current study, the clinical and technical expert team was employed to identify the needs and functional features of the decision support application. In the study of Karthikeyan et al., (2019) a total of 475 applications related to eye care were identified on the Android platform, yet ophthalmologists participated in the design and development of only 107 (22.53%) applications [39]. The design of clinical systems according to the opinion of users with different backgrounds helps to interpret and use the systems more easily and consequently enhance them [40, 41]; it is therefore recommended to use the opinions of both technical and clinical experts in designing these systems more fully from technical and clinical perspective.

In this study, retina fellows were interviewed to identify the user needs. In the present study, similar to the studies on the development of decision support applications (MELANI) for medicinal plants [42] and Parkinson's disease [43], and the development of decision support systems for Sleep Staging Tasks [44] and rare diseases [45], end users were interviewed to identify the needs and the features. In interviews with key users, experiences, attitudes and personal beliefs related to the subject of interest are determined [46]. Identifying the needs and features of the system in this way helps design the systems in the best way possible according to the needs of end users, which can affect usability by interested end users and the success of the system.

The multipurpose application in the present study, as with the clinical decision support monitoring and support systems in the study of Kart et al. (2017) [47] and the electronic tool designed in the study of Melnick et al., (2017) [48] was designed for use by both physicians and patients, which allows the sharing of clinical information among physicians and also between the physicians and patients. Meta-analyses [49, 50] have shown that CDSSs that are capable of providing recommendations to patients and specialists, are more effective than CDSSs that are only capable of providing recommendations to healthcare professionals. Besides this, multipurpose clinical decision support applications can facilitate their joint utilization by all authorized people in delivering patient care, which in turn can increase awareness, facilitate access to data, improve physician efficiency, and lead to more constructive communication between patients and healthcare teams [51, 52].

Ophthalmology residents rated this application as good through the usability evaluation and attained the highest score on the screen domain, which is consistent with the evaluation of the design and development of personal electronic health records for patients with thalassemia major [53] in the learning domain, as well as user interface of a smartphone-based application for increasing the self-care of patients with asthma [30]. Users rated this application as good, similar to the results of other studies [31, 54–57]. Given the importance of usability evaluation in the design and implementation of applications and identifying strengths and weaknesses [58], it is recommended that in future studies, the application be evaluated by patients to determine the problems related to the patient user interface.

### Study strengths and limitations

This study was the first to design, develop and evaluate an intelligent clinical decision support application for APE to assist the physicians in early diagnosis of the disease and also to help patients present to healthcare centers early. The strengths of the study included the active engagement of ophthalmologists and other specialists in the needs assessment and application design and adherence to evidence-based principles. Besides that, the use of artificial intelligence algorithms for more accurate prediction of disease diagnosis and the utilization of multimedia functions in the application are two other strengths of our study. One of the limitations of the study is the lack of sufficient time to interview with the physicians and hold focus group meetings, which was partly resolved by negotiating and making necessary coordination with them and conducting interviews when they were not present in the clinic. Model performance evaluation in our study using training data with an precision of 1 shows good performance in detection, but these results cannot be generalized to new data and do not represent the true performance of the model, as no system is 100% and should be checked with new data. The design phase in our study was done based on the opinions of the ophthalmologists of a teaching medical center and the lack of participation of patients in the design of the user interface was another limitation of the study, however, we did our best to design a user-friendly and practical application by interviewing all retina fellows and ophthalmology residents in this center, and to design the patient user interface based on the specialists' knowledge about patient needs and expectations.

## Conclusion

All-round participation and using the experiences of clinical specialists, and their awareness of patient needs, as well as the availability of comprehensive APE clinical dataset in the hospital led to the design of a system with accuracy, precision sensitivity, specificity, NPV, F-measure and AUC 100%. Since we reached the same results for all 4 algorithms, considering the volume and type of dataset, the decision tree algorithm was chosen for its simple interpretation and expressive quality for disease diagnosis in the decision support system. It is suggested that for the real evaluation of the performance of the model, new data for test should be used and the results determined using evaluation criteria. It is recommended to evaluate the extent of physician’s use of the application and how it affected the diagnosis of endophthalmitis in clinical settings in future studies.

## Supplementary Information

**Additional file 1:** **Table S1.** Interview guide.

**Additional file 2:** **Table S2.** Questionnaire to evaluate the satisfaction of users of the clinical decision support application for the diagnosis of endophthalmitis.

**Additional file 3:** **Table S3.** The results from interviews based on thematic analysis.

**Additional file 4:** **Figure S1. **Screenshots of the decision support application.

## Data Availability

The datasets used and analysed during the current study available from the corresponding author on reasonable request.

## Abbreviations

APE: Acute postoperative endophthalmitis
CDSSs: Clinical decision support systems
DR: Diabetic retinopathy
SVM: Support vector machine
DT: Decision tree
KNN: K-nearest neighbors
RF: Random forest
QUIS: Questionnaire for user interface satisfaction
ROC: Receiver operator characteristic
AUC: Area under precision-recall curve
SD: Standard deviation
DNNs: Deep neural networks
