# Supplementary material for: An intelligent decision support system for acute postoperative endophthalmitis: design, development and evaluation of a smartphone application
Source: BMC Med Inform Decis Mak. 2023 Jul 21;23:130. doi: 10.1186/s12911-023-02214-3 (PMC10362640; doi:10.1186/s12911-023-02214-3)
Supplement: Supplementary file 1 — Additional file 1: Table S1. Interview guide. [file 12911_2023_2214_MOESM1_ESM.docx]

**Interview guide**

Hi, my name is Azam Salehzadeh of Kashan University of Medical Sciences, and I need the help of ophthalmologists in Khatam-Al-Anbia Eye Hospital, Mashhad University of Medical Sciences to determine the needs and expectations of users and the content and features of the application.

I asked to talk to you because you are the ophthalmologist of the said center.
The information you share will help us to create better educational materials for physicians. I would like to record our conversation today and make some notes. The
recording will be typed out. Only researchers on the project will be able to review the recordings or any notes. The recordings and notes will be destroyed at the end of the study.

Please take a moment and look over the consent form. It states that:
--everything you say and all information we gather will be confidential; and the results of the study are published anonymously.
--your participation is voluntary, you may refuse to answer any question, and you may stop the
interview at any time.
Please give verbal assent to indicate you agree to participate and I record your informed consent.
Thank you.
I will now start recording. [Start recording. Questions are in bold.]
This interview should last no more than 120 minutes. I have several questions to ask, but I would
like you to do most of the talking and give as much detail as you can with your answers.

Choice of disease

**In your opinion, which disease is the challenge of emergency ophthalmology? [with the reason]**

**Does this disease have different types?**

**If the answer is yes, which type do you think is more suitable for use in the application?**

Determine the end users of the app

**Do you agree with assigning the application to the group of physicians? [with the reason]**

**If the answer is yes, in your opinion, in which stage of the disease (diagnosis or treatment) can the app be more useful for physicians?**

**Do you agree with allocating part of the application to patients? [with the reason]**

**If the answer is yes, how do you think the app can be useful for patients?**

Determine the type of app language

**What is your suggestion for the type of app language for physicians?**

**[Choose applicable one]**

€ Persian
€ English

€ both

**What is your suggestion for the type of app language for patients?**

**[Choose applicable one]**

€ Persian
€ English

€ both

**What is your opinion about how to design questions for physicians and patients?**

Functional requirements

**From the list of functional requirements and the list of clinical symptoms prepared based on the review of the literature provided to you, which requirement and which clinical symptom do you approve or remove for inclusion in the app?**

App resources

**The application can be used based on what sources?**

**Is it necessary to have resources in the app?**

Reporting of actions taken

**Is it necessary to register the activity time in the app? [with the reason]**

**Is it necessary to record the actions taken in the app? [with the reason]**

Determining the patient's medical history and drug use

**What is the best way to include the list of diseases and the list of medicines in the disease records section of the app?**

**Is it important to determine the amount of medication prescribed in the app?**

**If the answer is yes, state the drugs related to the disease and the appropriate dosage.**

The ability to share information and the ability to interact with the patient and the physician

**Do you agree to share physician-to-physician/patient-to-physician information on the app? [with the reason]**

**Is it possible to ask and answer common questions of patients and physicians in the application? [with the reason]**

Determining treatment recommendations

**Is it necessary to provide treatment recommendations in the app? If you agree, explain how these recommendations and actions in app should be in the app?**

Ability to upload image and play sound

**Do you agree to use audio and video technologies for the use of physicians and patients in the app?**

Can you think of anything else that would be helpful to you or other patients who are in
this situation?
I have no more planned questions. But is there anything else you would like to add?

Thank you very much for allowing me to interview you. We are very hopeful that the information
that you share can be used to help future physicians and patients faced with this disease.
