# Supplementary material for: An intelligent decision support system for acute postoperative endophthalmitis: design, development and evaluation of a smartphone application
Source: BMC Med Inform Decis Mak. 2023 Jul 21;23:130. doi: 10.1186/s12911-023-02214-3 (PMC10362640; doi:10.1186/s12911-023-02214-3)
Supplement: Supplementary file 2 — Additional file 2: Table S2. Questionnaire to evaluate the satisfaction of users of the clinical decision support application for the diagnosis of endophthalmitis. [file 12911_2023_2214_MOESM2_ESM.docx]

**Questionnaire to evaluate the satisfaction of users of the clinical decision support application for the diagnosis of endophthalmitis**

Greetings and Regards,

One of the stages of designing and implementing the "Clinical Decision Support Application for the Diagnosis of Endophthalmitis" is the usability test, and the questionnaire you are facing is designed to evaluate the usability and satisfaction of users in relation to the use of this application. This questionnaire contains 27 questions. After reading each question, please enter the corresponding answer in one of the 10 boxes opposite. Choosing a larger number indicates more capabilities of the application and your level of satisfaction with the application and its features, and a smaller number means low capabilities and your level of satisfaction with this application. The results of this review will be used to make additional corrections and improve this application.

**A: Personal characteristics of physicians**

| Age (year): .............................................. | Gender: Female Male |
| --- | --- |
| Place of service (university/hospital): ..................................... | Medical experience (years): .............................................. |

**B: After reading the following items, please mark your comments in the form of the answers provided: (0 indicates the worst situation and 9 indicates the best situation)**

| No. | Title | Response | 0 | 1 | 2 | 3 | 4 | 5 | 6 | 7 | 8 | 9 | Response |
| --- | --- | --- | --- | --- | --- | --- | --- | --- | --- | --- | --- | --- | --- |
| **Overall Reaction To The Software** | | | | | | | | | | | | | |
| 1 | terrible | |  |  |  |  |  |  |  |  |  |  | wonderful |
| 2 | difficult | |  |  |  |  |  |  |  |  |  |  | easy |
| 3 | frustrating | |  |  |  |  |  |  |  |  |  |  | satisfying |
| 4 | Inadequate power | |  |  |  |  |  |  |  |  |  |  | adequate power |
| 5 | dull | |  |  |  |  |  |  |  |  |  |  | stimulating |
| 6 | rigid | |  |  |  |  |  |  |  |  |  |  | flexible |
| **SCREEN** | | | | | | | | | | | | | |
| 7 | Characters on the computer screen | hard to read |  |  |  |  |  |  |  |  |  |  | easy to read |
| 8 | Highlighting on the screen simplifies task | not at all |  |  |  |  |  |  |  |  |  |  | very much |
| 9 | Organization of information on screen | confusing |  |  |  |  |  |  |  |  |  |  | very clear |
| 10 | Sequence of screens | confusing |  |  |  |  |  |  |  |  |  |  | very clear |
| **TERMINOLOGY AND SYSTEM INFORMATION** | | | | | | | | | | | | | |
| 11 | Use of terms throughout system | inconsistent |  |  |  |  |  |  |  |  |  |  | consistent |
| 12 | Computer terminology is related to the task you are doing | never |  |  |  |  |  |  |  |  |  |  | always |
| 13 | Position of messages on screen | inconsistent |  |  |  |  |  |  |  |  |  |  | consistent |
| 14 | Messages on screen which prompt user for input | confusing |  |  |  |  |  |  |  |  |  |  | clear |
| 15 | Computer keeps you informed about what it is doing | never |  |  |  |  |  |  |  |  |  |  | always |
| 16 | Error messages | unhelpful |  |  |  |  |  |  |  |  |  |  | helpful |
| **LEARNING** | | | | | | | | | | | | | |
| 17 | Learning to operate the system | difficult |  |  |  |  |  |  |  |  |  |  | easy |
| 18 | Exploring new features by trial and error | difficult |  |  |  |  |  |  |  |  |  |  | easy |
| 19 | Remembering names and use of commands | difficult |  |  |  |  |  |  |  |  |  |  | easy |
| 20 | Tasks can be performed in a straight  -forward manner | never |  |  |  |  |  |  |  |  |  |  | always |
| 21 | Help messages on the screen | unhelpful |  |  |  |  |  |  |  |  |  |  | helpful |
| 22 | Supplemental reference materials | confusing |  |  |  |  |  |  |  |  |  |  | clear |
| **SYSTEM CAPABILITIES** | | | | | | | | | | | | | |
| 23 | System speed | too slow |  |  |  |  |  |  |  |  |  |  | fast enough |
| 24 | System reliability | unreliable |  |  |  |  |  |  |  |  |  |  | reliable |
| 25 | System tends to be | noisy |  |  |  |  |  |  |  |  |  |  | quiet |
| 26 | Correcting your mistakes | difficult |  |  |  |  |  |  |  |  |  |  | easy |
| 27 | Experienced and inexperienced users’ needs are taken into consideration | never |  |  |  |  |  |  |  |  |  |  | always |
