# Supplementary material for: An intelligent decision support system for acute postoperative endophthalmitis: design, development and evaluation of a smartphone application
Source: BMC Med Inform Decis Mak. 2023 Jul 21;23:130. doi: 10.1186/s12911-023-02214-3 (PMC10362640; doi:10.1186/s12911-023-02214-3)
Supplement: Supplementary file 3 — Additional file 3: Table S3. The results from interviews based on thematic analysis. [file 12911_2023_2214_MOESM3_ESM.docx]

**Table S3** The results from interviews based on thematic analysis

| **Main theme** | **Subtheme** | **Concept (initial code)** |
| --- | --- | --- |
| Needs and expectations | Selecting (predicting) disease | Problem with disease diagnosis |
|  |  | Determining the type of disease used in the application |
|  | Determining the end users of the application  (physicians and patients) | Dedicating a part of the application to patients |
|  |  | Dedicating the application to the team of physicians |
|  | Determining the type of application language  (For physician: English  For the patient: Persian) | Using English concepts and assigning English language for physicians in the application |
|  |  | The type of question is understandable and simple, and assign Persian language for patients in the application |
| Functional features of the application | Creating resources in the application | Giving access to clinical information, guidelines and authoritative and reference books for physicians |
|  |  | Providing information needed by patients |
|  | Creating reports of activities | Recording the activity time in the application |
|  |  | Recording activities in the application |
|  | Determining patient medical and medication history | Inserting the list of diseases in the application for medical history |
|  |  | Inserting the list of medications in groups in the application for medication history |
|  | Selecting the appropriate treatment | Determining the dose of prescribed drug in the application |
|  |  | Selecting appropriate treatments as the main treatment and supportive treatment |
|  | Sharing information and  physician-patient communication | Sharing patient information with the physician in the application |
|  |  | Frequently Asked Questions for patients and physicians |
|  | Uploading images and playing audio files for patients | Creating an audio play feature for ease of use by patients |
|  |  | Uploading images by patient in the application |
