# Supplementary material for: An intelligent decision support system for acute postoperative endophthalmitis: design, development and evaluation of a smartphone application
Source: BMC Med Inform Decis Mak. 2023 Jul 21;23:130. doi: 10.1186/s12911-023-02214-3 (PMC10362640; doi:10.1186/s12911-023-02214-3)
Supplement: Supplementary file 4 — Additional file 4: Figure S1. Screenshots of the decision support application. [file 12911_2023_2214_MOESM4_ESM.docx]

**(A-F) Screenshots of the decision support application**


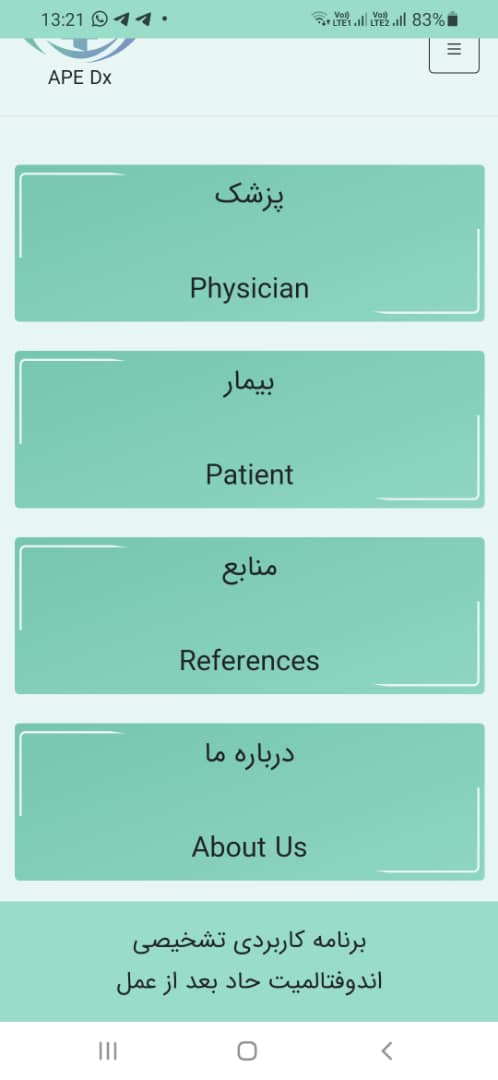


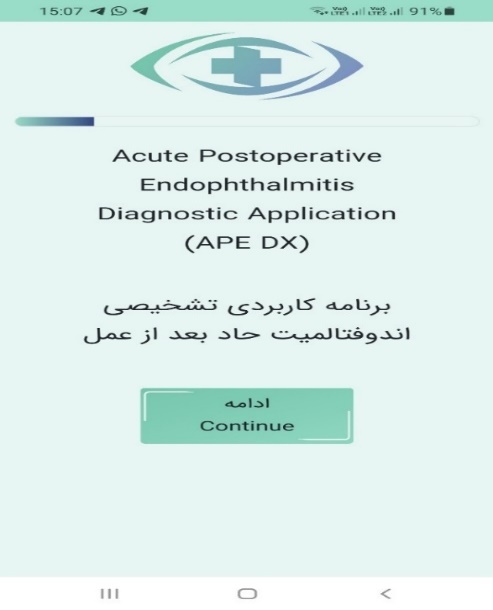


A. Login page B. Home page


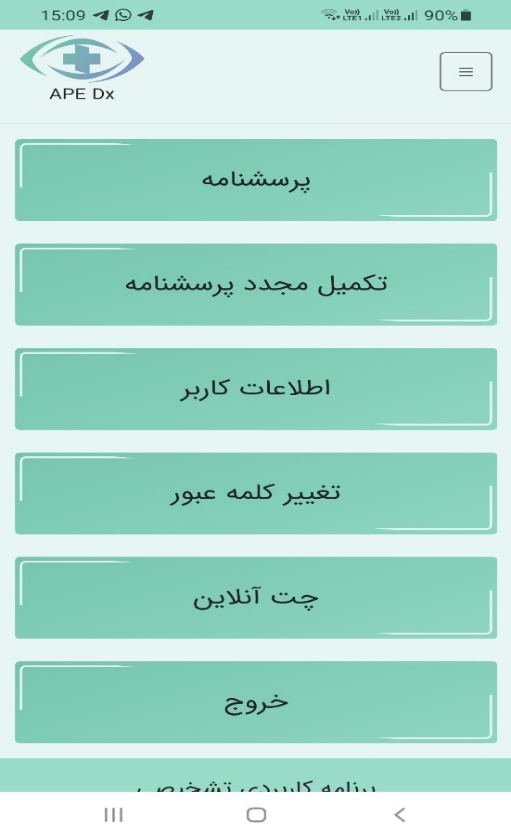

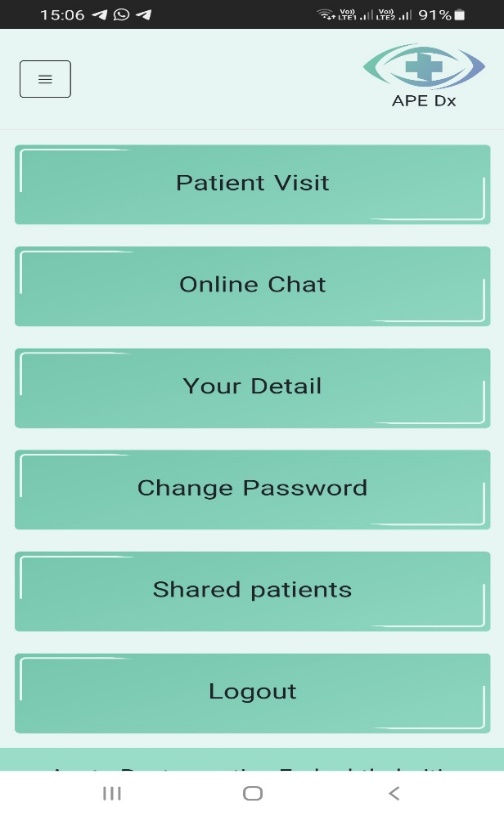
C. Physician user interface D. Patient user interface


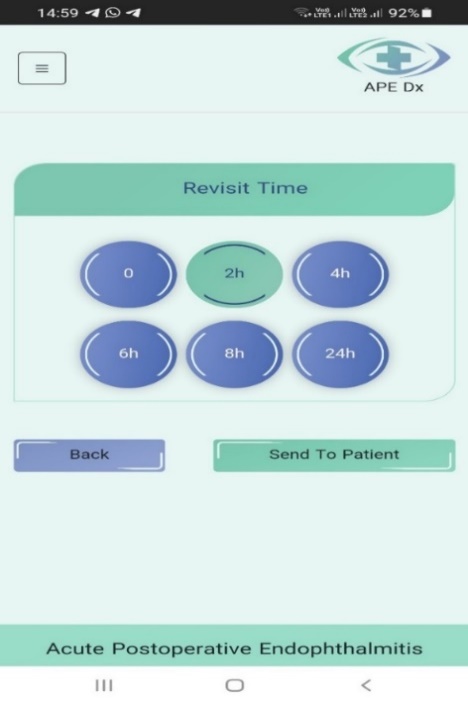

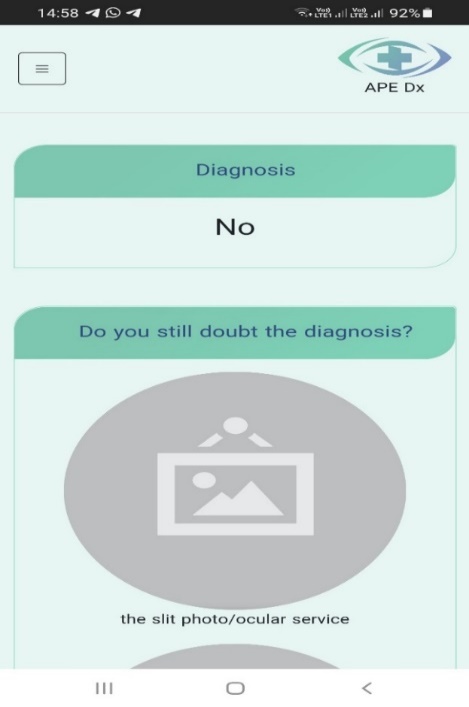

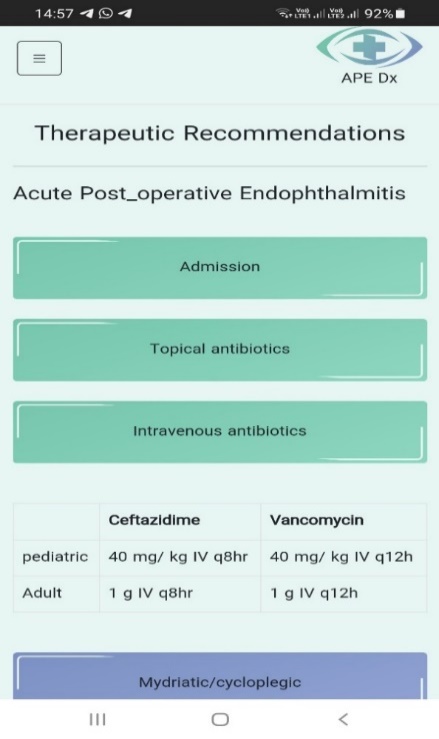


E1

E2

E3

E. Sample screenshots from physician user interface, E1) therapeutic recommendation, E2) diagnosis, E3) revisit time/ fill out the questionnaire


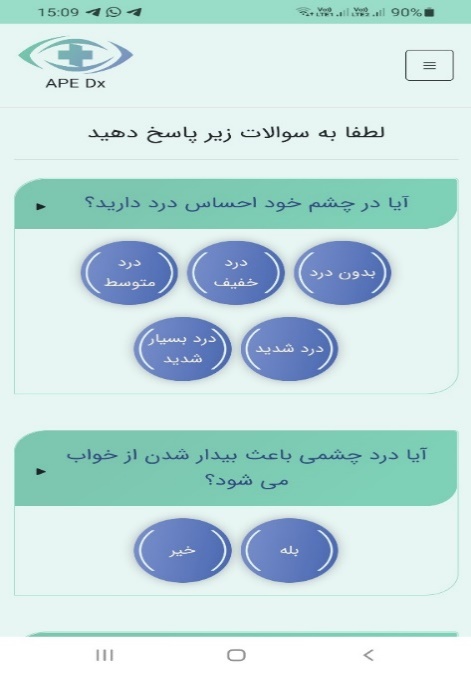

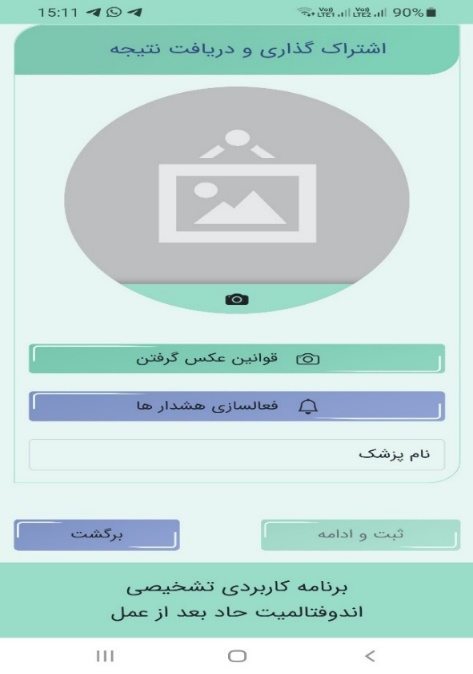


F2

F1

F. Sample screenshots from patient user interface, F1) Patient questionnaire, F2) Information sharing
